# A high throughput tensile ice adhesion measurement system

**DOI:** 10.1016/j.ohx.2020.e00146

**Published:** 2020-10-05

**Authors:** Kiana Mirshahidi, Kamran Alasvand Zarasvand, Wenting Luo, Kevin Golovin

**Affiliations:** Okanagan Polymer Engineering Research & Applications Laboratory, School of Engineering, University of British Columbia, Kelowna, BC, Canada

**Keywords:** Tensile ice adhesion system, Interfacial cavitation, Tension-mediated fracture, Tensile adhesion measurement device, Icephobic

## Abstract

A prerequisite for designing materials with low adhesion to ice is to accurately measure the ice adhesion strength of the surface. The majority of studies in this field have typically focused on manipulating and measuring the adhesion strength of different materials under shear stress. Among them, elastomers have proven to be promising ice-phobic surfaces because they enable interfacial cavitation, a tension-driven surface instability. In this work, a high throughput, low cost device is designed to measure the tensile ice adhesion strength of different surfaces. The design and construction of the tensile ice adhesion measurement system is presented, along with the reasoning for the design decisions. The performance of the setup is characterized using experimental trials varying parameters such as temperature, pull-off speed, thickness of the substrate, and ice/substrate interfacial area, to verify the precision of the measurements.

## Graphical abstract

**Figure:**
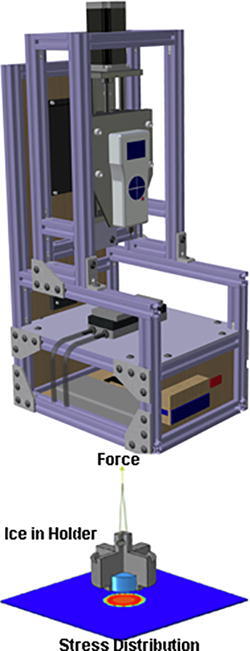

Specifications table:

**Table:** 

Hardware name	Tensile Ice Adhesion Measurement System
Subject area	• Engineering and Material Science
Hardware type	• Measuring physical properties and in-lab sensors • Mechanical engineering and materials science
Open Source License	CC BY-SA 3.0
Cost of Hardware	$1,450 with sink access or $2,000 closed-loop system
Source File Repository	

## 1 Hardware in context

The accretion of ice can cause significant issues and can damage the iced structure. Regular removal of the ice can lead to further damage, mainly due to the strong adhesion between ice and most materials. To overcome this issue many scientists have successfully designed and fabricated surfaces with reduced adhesion to ice [1], [2]. Elastomeric films with ultra-low adhesion to ice have recently been developed [3], [4], [5], [6]. The low ice adhesion is enabled by a surface instability known as interfacial cavitation, which is a tension-driven phenomenon [7]. However, most ice adhesion measurement devices can only measure the shear ice adhesion strength of surfaces [8], [9].

There is no standard or high throughput system readily available, and few studies have utilized devices and methods that measure the adhesion of ice by applying a tensile force [10], [11], [12], [13], [14], [15], [16], [17], [18], [19], [20], [21], [22], [23], [24], [25], [26]. Among them, Yan et al. [27] used a universal mechanical testing machine to measure the tensile adhesion of ice by placing the entire measurement device into a freezing room in order to produce the ice. To measure the adhesion they removed the device from the freezing room to fix the upper handle on the universal mechanical testing machine. This method increases the risk of environmental error due to temperature change during sample transport and handling. A further disadvantage of this method is that only one sample can be tested during each run. Another method that has been used to detach ice in the direction normal to the surface is the so-called blister method. Here pressurized air is pumped into a cylindrical crack from beneath the ice/surface interface. Davis et al. [28] used this method to measure the adhesion of ice to their surfaces with different values of roughness. However, this device is quite complex, requires a starter crack, and has only been demonstrated inside an icing wind tunnel, which are rare and expensive to fabricate.

Here, we discuss the design and construction of a tensile ice adhesion measurement device which exhibits many advantages over previous measurement systems. The benchtop system has a small footprint (overall dimensions of 26 cm × 33 cm × 70 cm, length × width × height) and is simple to construct and operate. The device mainly consists of a motor, force gauge, cold stage, ice holders, chiller, and aluminum framing which provides mechanical support to the structure. A thermocouple and a PID controller regulate the temperature of the Peltier stage where measurement takes place. Various experimental test parameters such as temperature, speed of the applied force, iced area, and thickness of the test material can be varied independently.

### 1.1 Hardware description

Unlike a typical uniaxial tension setup, our tensile ice adhesion setup includes a Peltier stage with an area of 12 cm × 6 cm, which also serves as the measurement stage. Larger Peltier stages could be substituted to allow for a greater number of tested samples per freeze cycle. The temperature of the stage is tunable in the range of 0 °C to −25 °C using a thermocouple and a cold-water circulation system. This makes it possible to measure the tensile adhesion of ice to substrates at different temperatures. The size of the ice holder affects the number of measurements per freeze cycle. For example, the ice holder that was used to verify the operation of the setup under different temperatures, pull-off speeds, and thicknesses had an outer radius of 6 mm, allowing for 6 tensile ice adhesion measurements during each freeze cycle. Making several measurements during a single freeze cycle decreases the risk of errors due to varied testing conditions, resulting in more precise and consistent values.

Unlike previous tensile measurement systems [25], [26], [27], the freezing cycle and the tensile measurement occur on the same stage, meaning the ice/substrate sample doesn’t need to be carried to the measurement stage. Environmental errors such as ice surface melting are avoided. The apparatus allows for three degrees of freedom in the x-, y-, and z-directions. The x- and y- translation enables the operator to freely position the force gauge hook directly above an ice holder. The z-direction translation, which is essential for the tensile force measurement, is enabled by mounting the force gauge on a linear motion stage connected to a motor. The force gauge can be easily and quickly replaced by another with a larger or smaller load cell, such that surfaces with very different adhesion values may be investigated. To control the iced area, holders with specific internal surface areas were designed. The geometry and shape of the holders were optimized to achieve a uniform stress distribution at the interface. The ice holders are one the most influential elements that govern the precision of the measurements. Accordingly, the design and optimization of the ice holder is discussed below, in detail.

This device:

• Measures the tensile force needed to detach ice from a substrate
• Is inexpensive and simple to build
• Can measure several samples during a single freeze cycle
• Was verified and the measurements matched known laws of fracture mechanics

### 1.2 Design and optimization of the ice holder

**Fig. 1:**
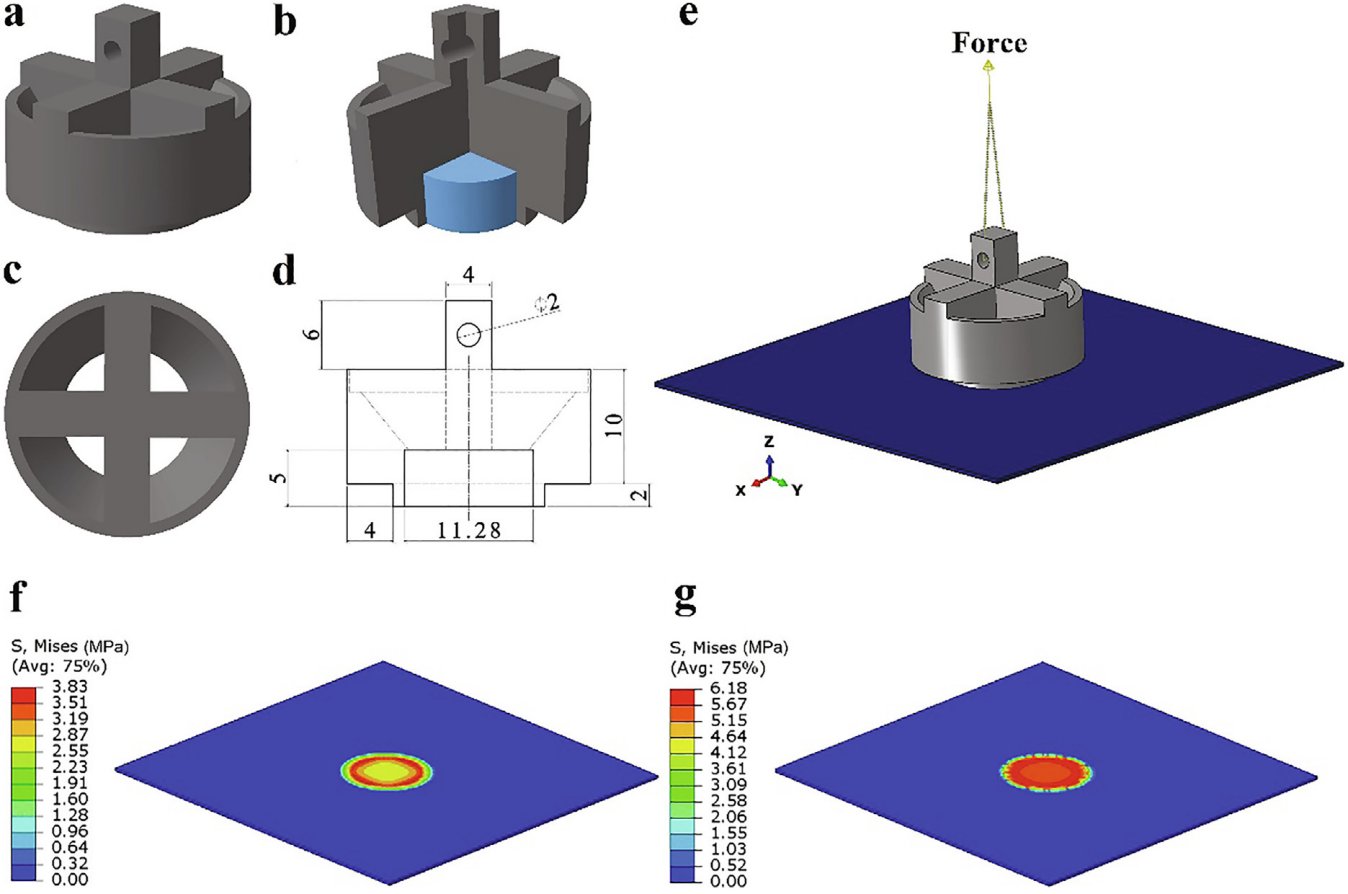
Tensile adhesion ice holder. **a,** Isometric view of the designed ice holder. **b,** Sectioning a quarter of the model reveals the cylindrical ice inside the holder. **c,** Two-dimensional top view of the ice holder. **d,** Drawing of designed ice holder (front view). The dimensions are in mm. **e,** Assembly model of the Mode I ice adhesion test. A concentrated force was applied to the connection points of the beam connectors via a high-strength cord, and the stress was transferred uniformly to the ice holder and ice adhered to the aluminum sheet. **f-g,** Stress distribution within the aluminum sheet. Uniform stress distribution was observed in the first (f) and second (g) increment (before separation) of the analysis.

Unlike shear-based methods, the design of the ice holder was more complex as tension must be applied normal to the interfacial plane. An ice holder was designed and analyzed using finite element modeling (Abaqus) in order to minimize stress concentration and avoid any induced bending moment. In contrast to the push-off shear ice adhesion test method, where uniform compression is applied on the lateral side of the ice cube holder, a pull-off method was simulated in order to investigate ice adhesion in Mode I fracture (tensile detachment). Uniformity in the stress distribution at the interface was considered in the optimized design of the ice holder, along with a methodology for easily and repeatedly applying tension. The deformable solid parts of the ice holder, cylindrical ice, and an aluminum substrate were modeled. In these simulations, the ice holder (made of polylactic acid, PLA, a common 3D-printing resin) was placed on an aluminum sheet with dimensions *L* = 60 mm, *W* = 60 mm, and *t* = 0.5 mm. The elastic moduli of aluminum, PLA, and ice were set to 70, 3.5, and 8.5 GPa, respectively, with Poisson ratios of 0.3, 0.33, and 0.3, respectively. The static analysis step was defined as ‘solver’ for all numerical simulations. After several iterations, the ice holder with the geometry shown in Fig. 1a-e was selected.

Interfacial fracture was modeled by applying a cohesive zone model to the ice/aluminum interface [29]. The values of the cohesive zone parameters obtained by Chen et al. [23] were utilized (Table 1). The interaction between the ice and ice holder was considered as a tie constraint to stipulate deformation continuity at the interfacial plane. The designed ice holder could effectively transfer the concentrated external force uniformly over the top surface of the cylindrical ice due to the chosen geometry (Fig. 1b). Undeformable beam connector elements were selected for the connection between the ice holder and loading points (Fig. 1e). These elements transferred the applied force between the components with a constrained component of relative motion (CORM) of the translational and rotational displacements in all three directions.

**Table 1:** Cohesive zone model parameters used in the numerical simulations [23].

Parameter	Values
Normal cohesive stiffness (*K_n_*)	1 × 10^6^N/mm^3^
Shear cohesive stiffness (*K_t_*)	1 × 10^6^N/mm^3^
Normal mode fracture energy (*G_cn_*)	1 × 10^−3^N/mm
Shear mode fracture energy (*G_ct_*)	1 × 10^−3^N/mm
Normal cohesive traction (*T_n_^Max^*)	0.8 MPa
Shear cohesive traction (*T_t_^Max^*)	0.8 MPa

**Fig. 2:**
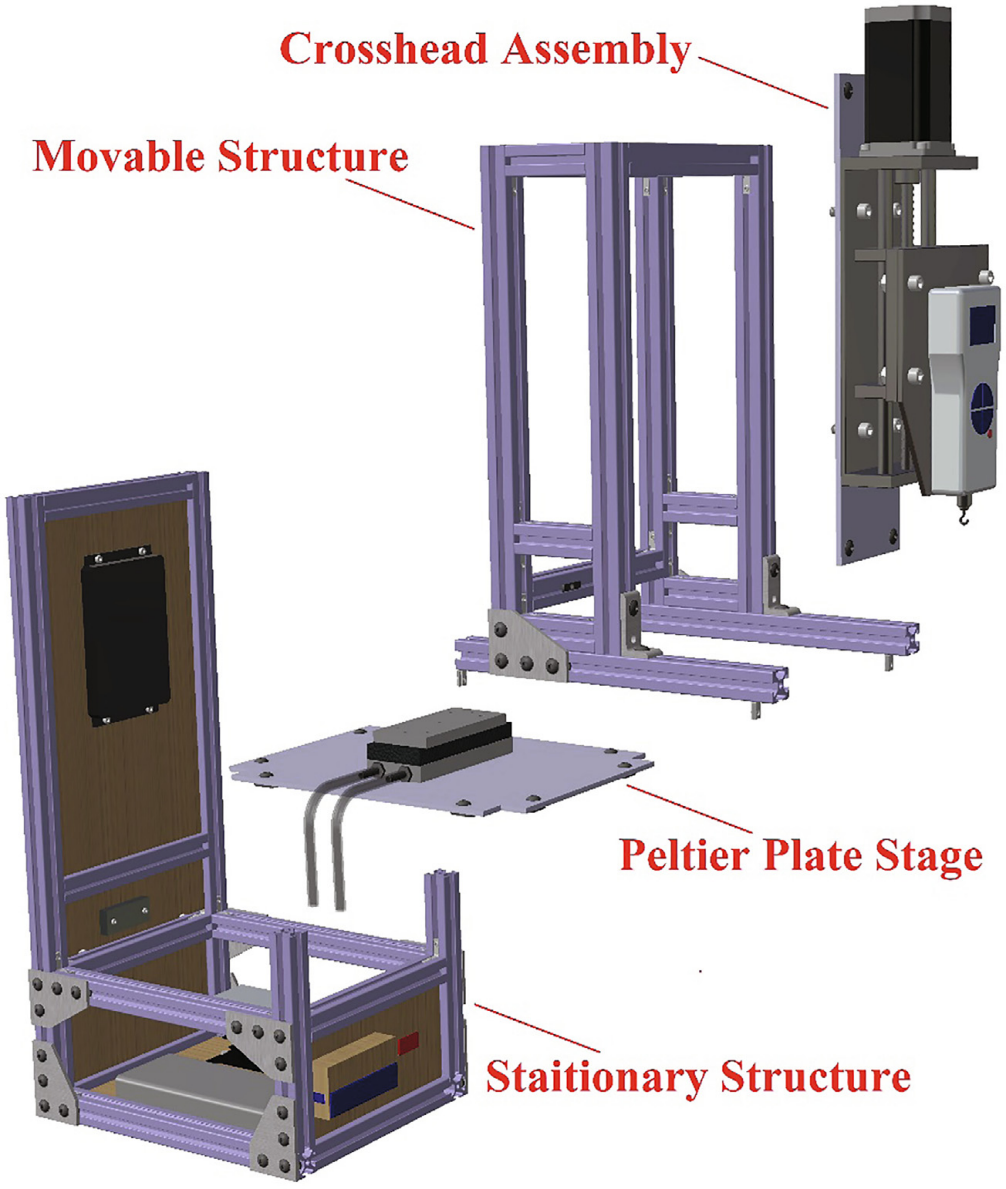
Tensile ice adhesion measurement system break-down into four main structures.

In order to optimize the element size in the numerical simulations, mesh convergence was studied. 14,400, 9,724 and 33,086 elements with type C3D8R, consisting of an 8-node linear brick with reduced integration, were used for the sheet, ice cube, and ice holder models, respectively. No significant variation in the maximum stress was observed in the aluminum sheet for a larger number of elements, and the optimum element size of 0.5 mm was selected. Overall, the relatively uniform stress distribution at the ice/aluminum interface (Fig. 1f,g) indicated that the designed ice holder should exhibit consistent tensile ice adhesion measurements.

**Fig. 3:**
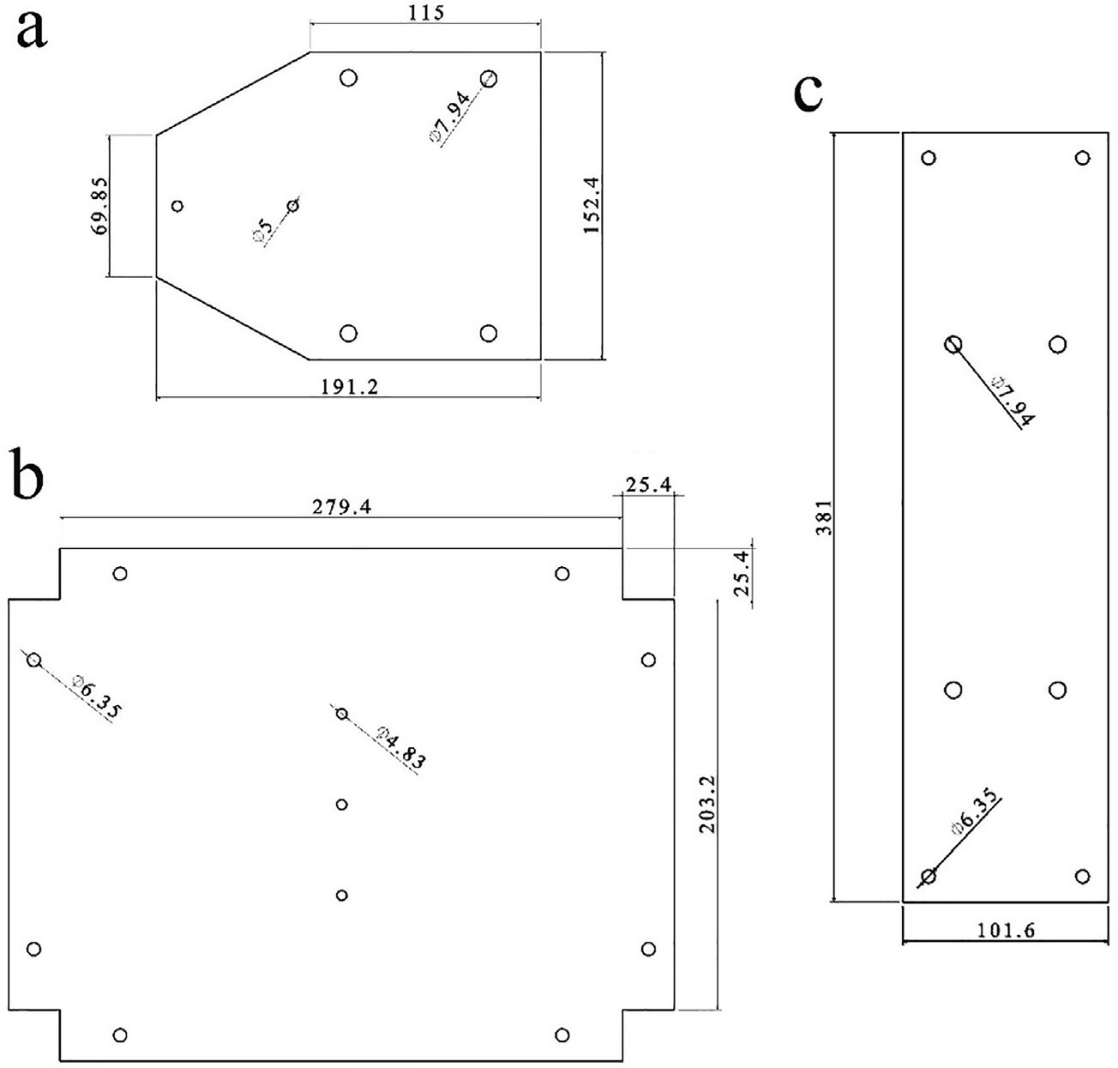
Dimensions and specifications of the aluminum sheets. **a,** Part (13) **b,** Part (26) **c,** Part (9). The dimensions are in mm.

## 2 Design files

### 2.1 Design files summary

**Fig. 4:**
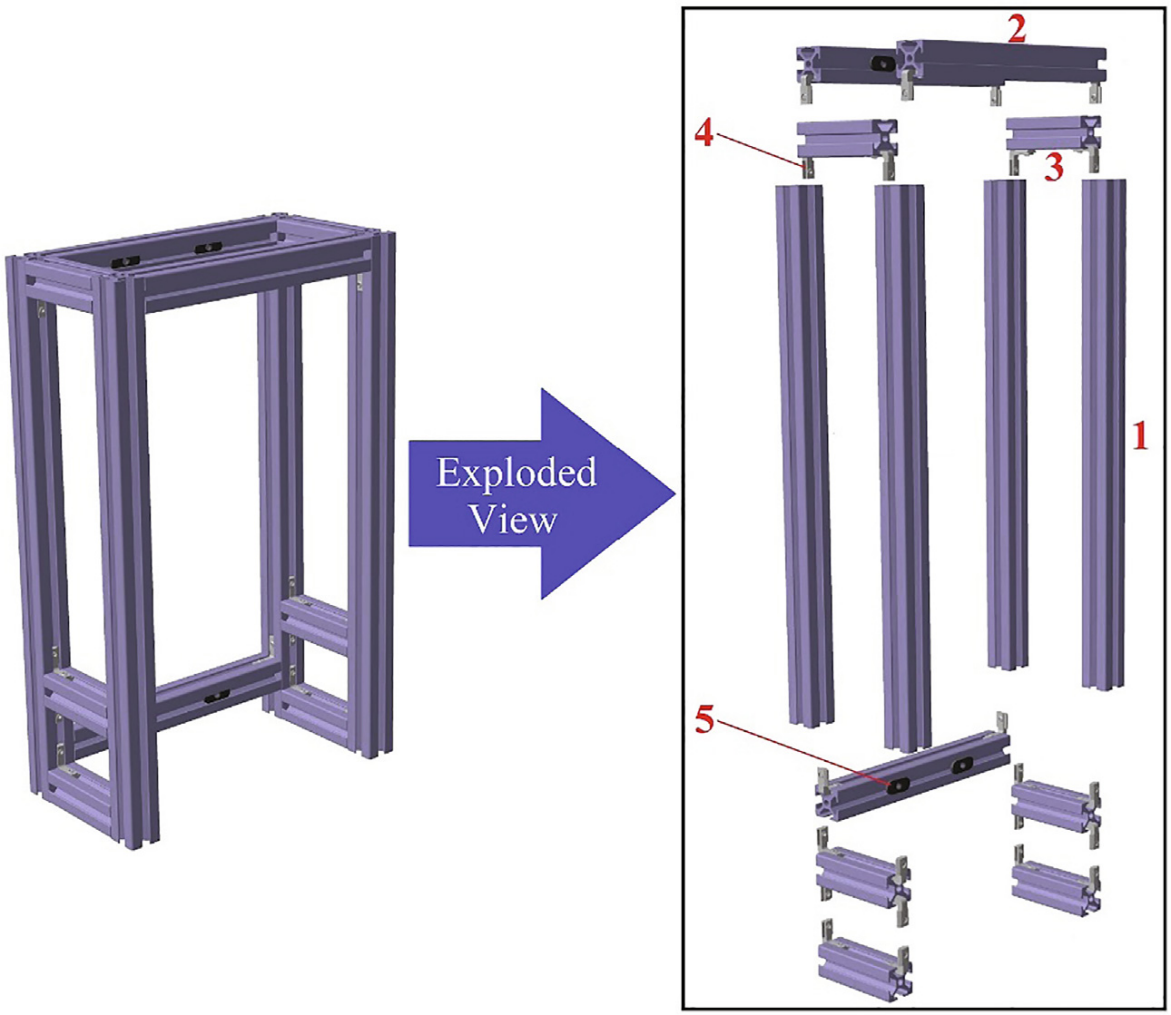
Movable structure framing completed view and exploded view.

**Fig. 5:**
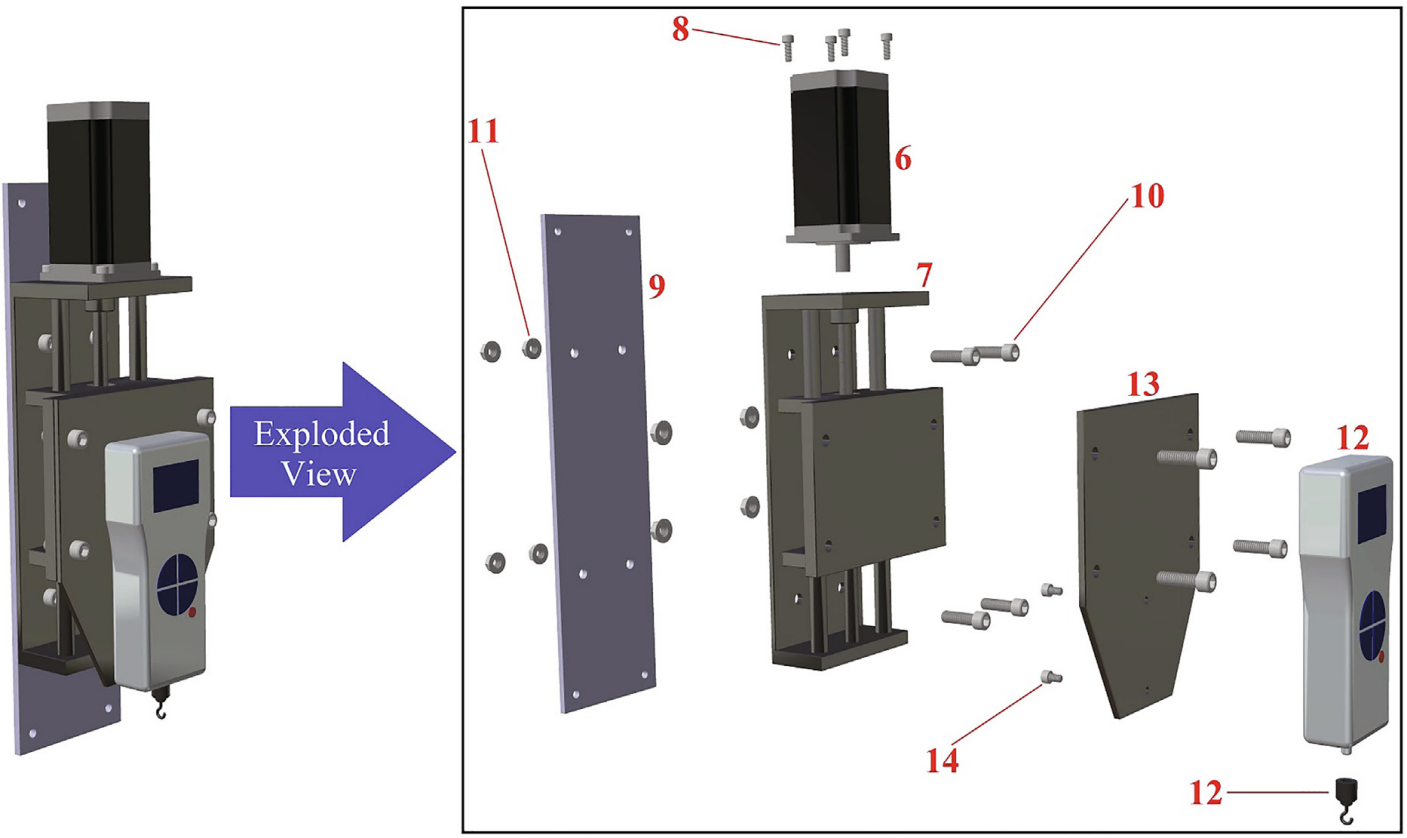
Crosshead assembly completed view and exploded view.

**Fig. 6:**
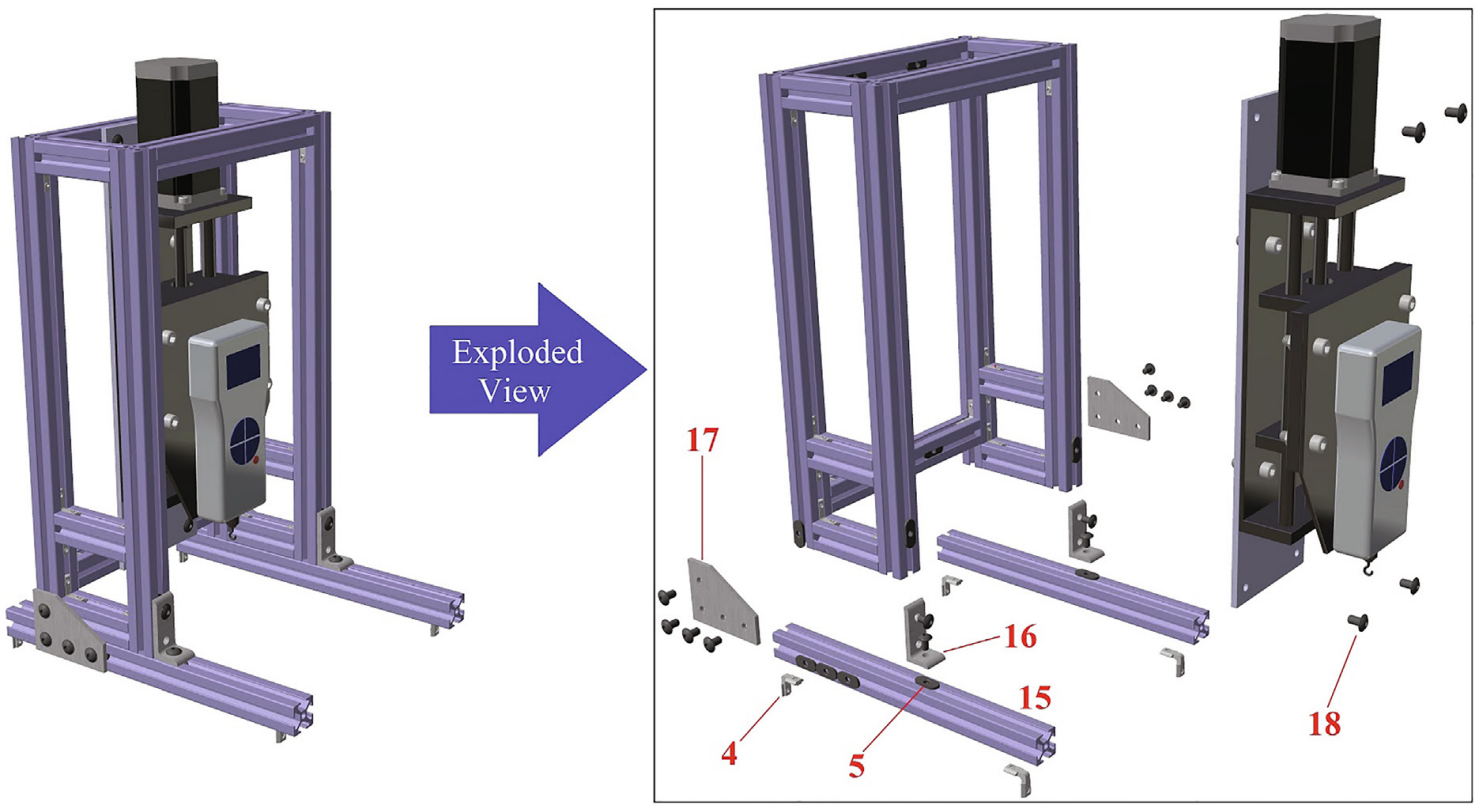
Crosshead assembly fixed on the movable structure, both completed view and exploded view.

**Fig. 7:**
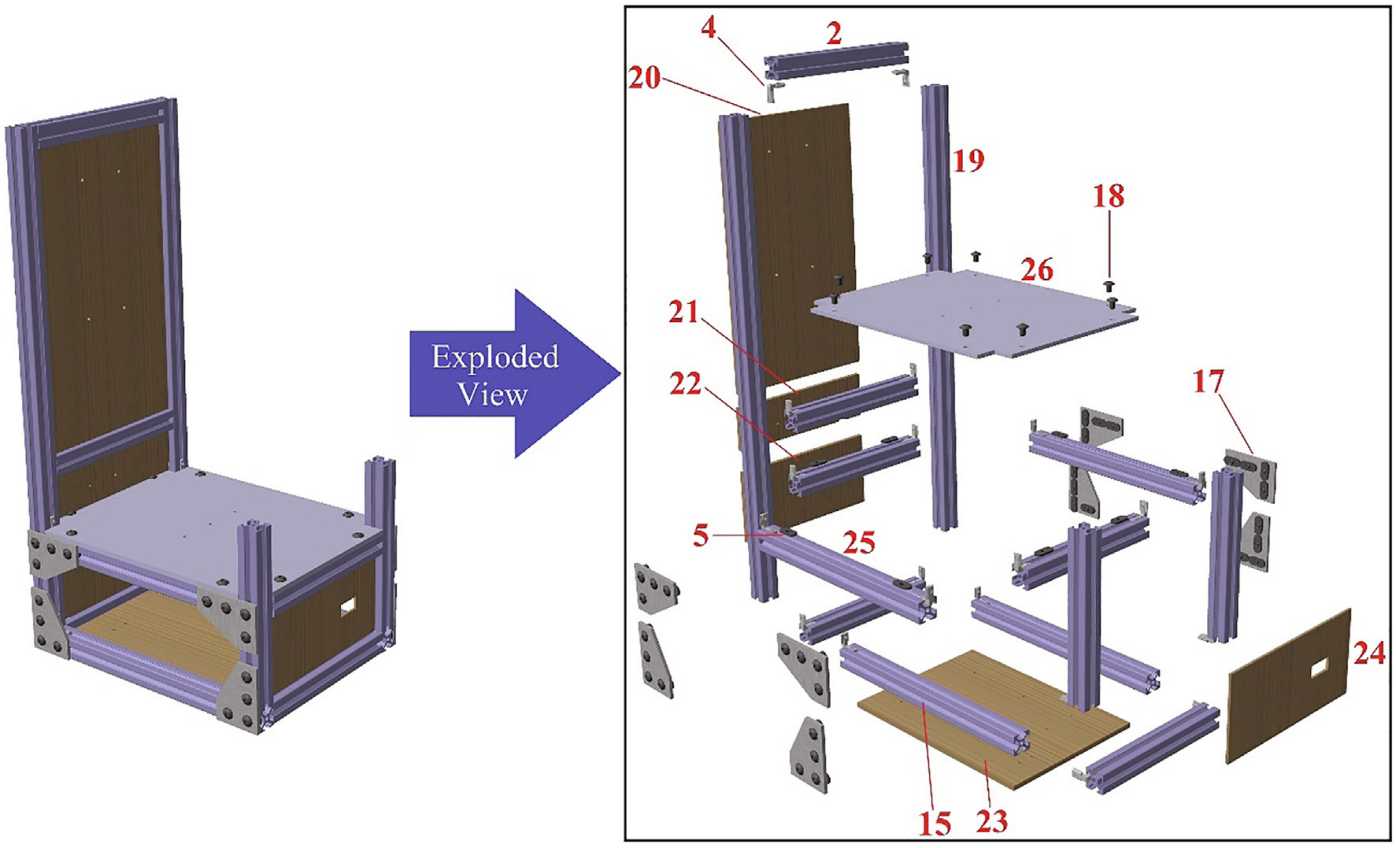
Stationary structure completed view and exploded view.

**Fig. 8:**
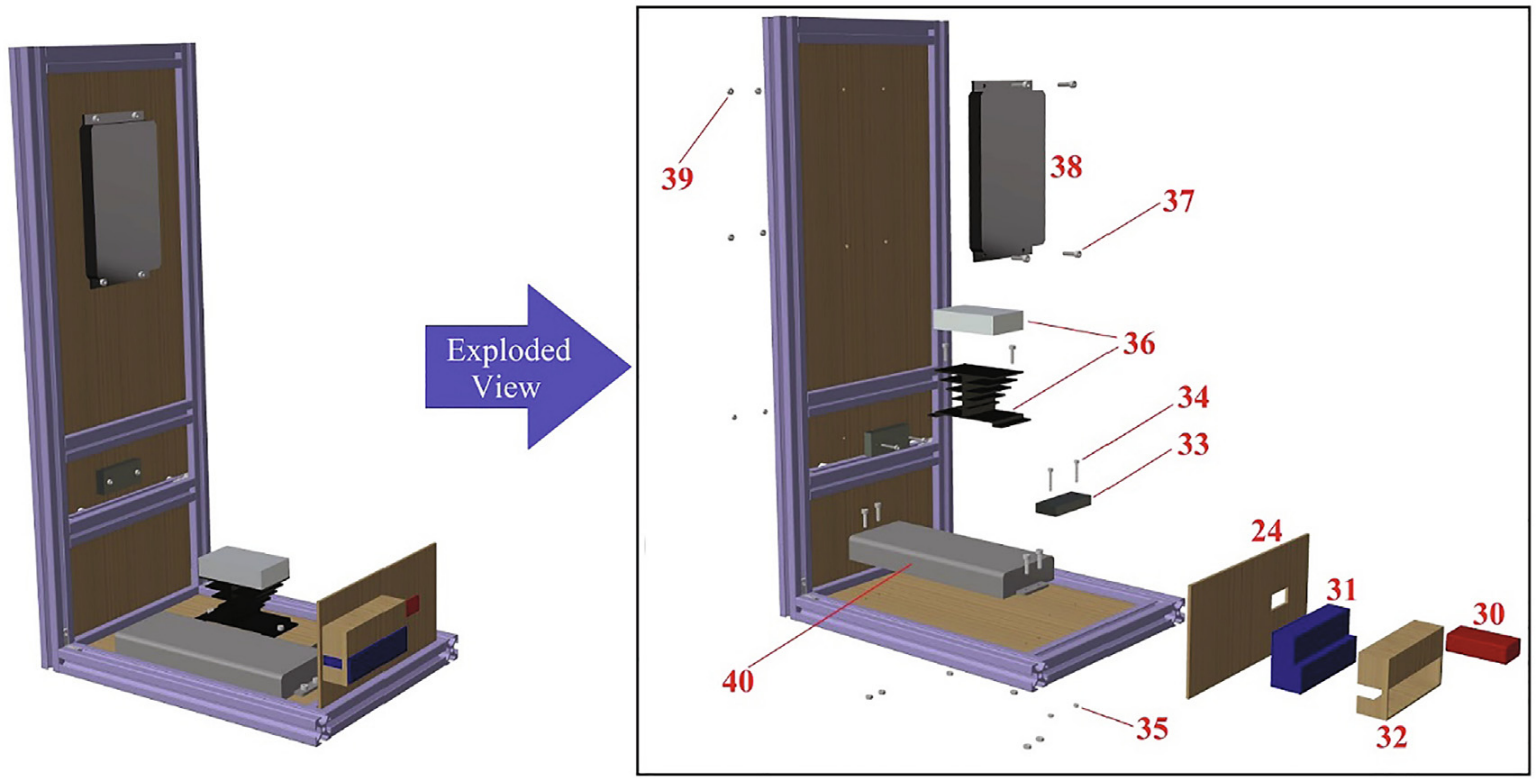
Installation of the electronics on the stationary structure.

**Fig. 9:**
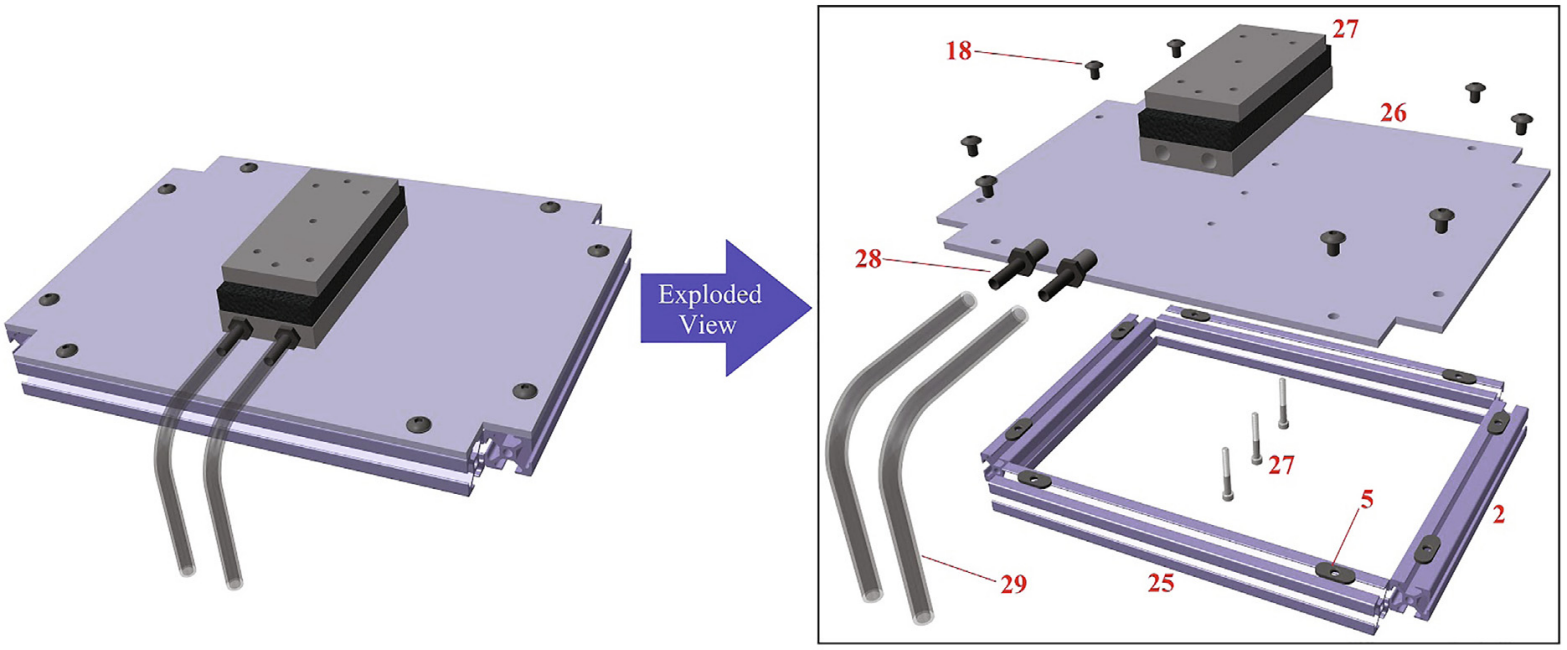
Peltier plate stage assembly, both completed view and exploded view. Note that (27) comes with 3 internal screws that are removed and re-attached to fix (27) onto (26).

**Fig. 10:**
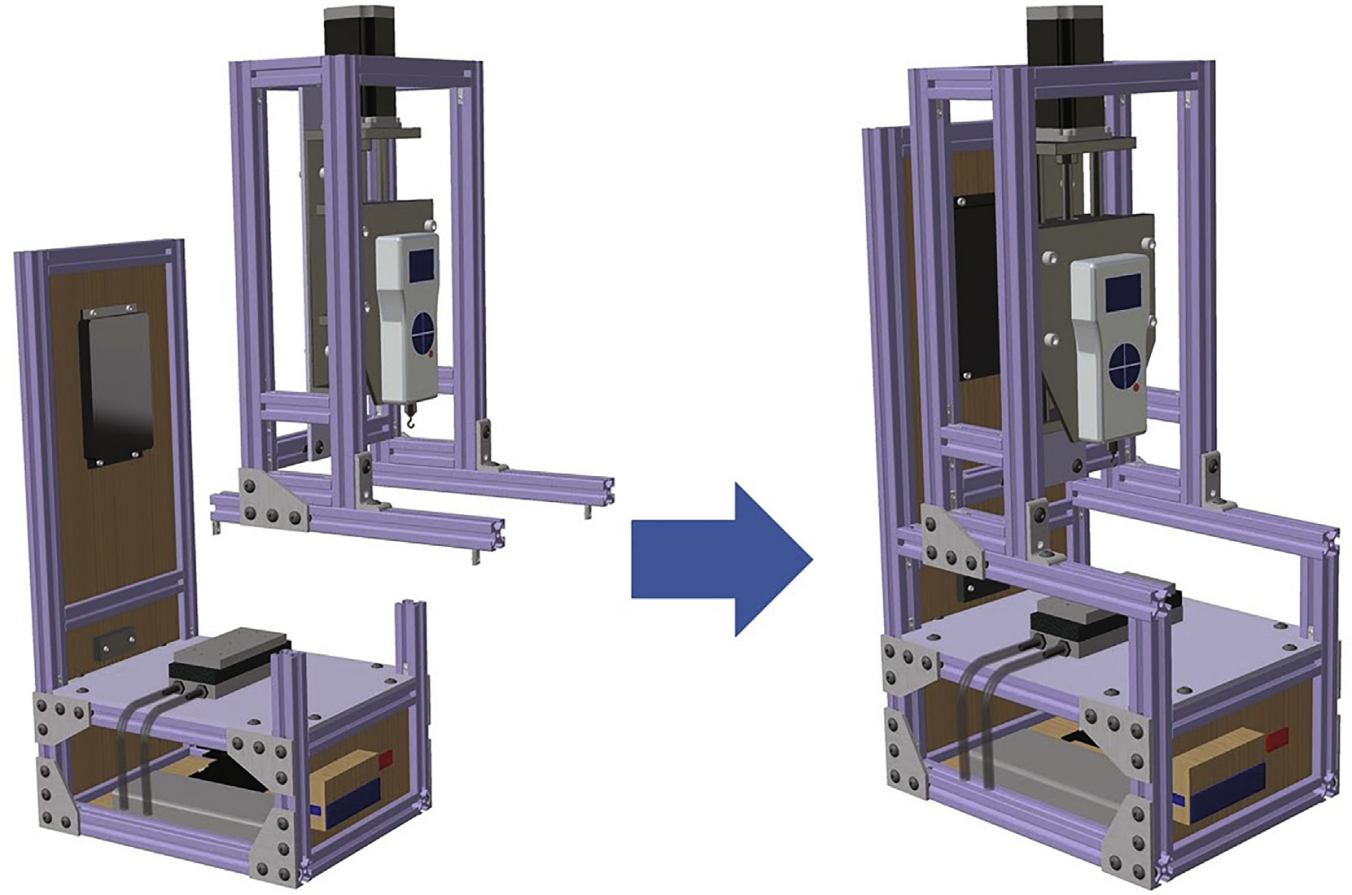
The Peltier plate stage and movable structure fixed on the stationary structure.

**Fig. 11:**
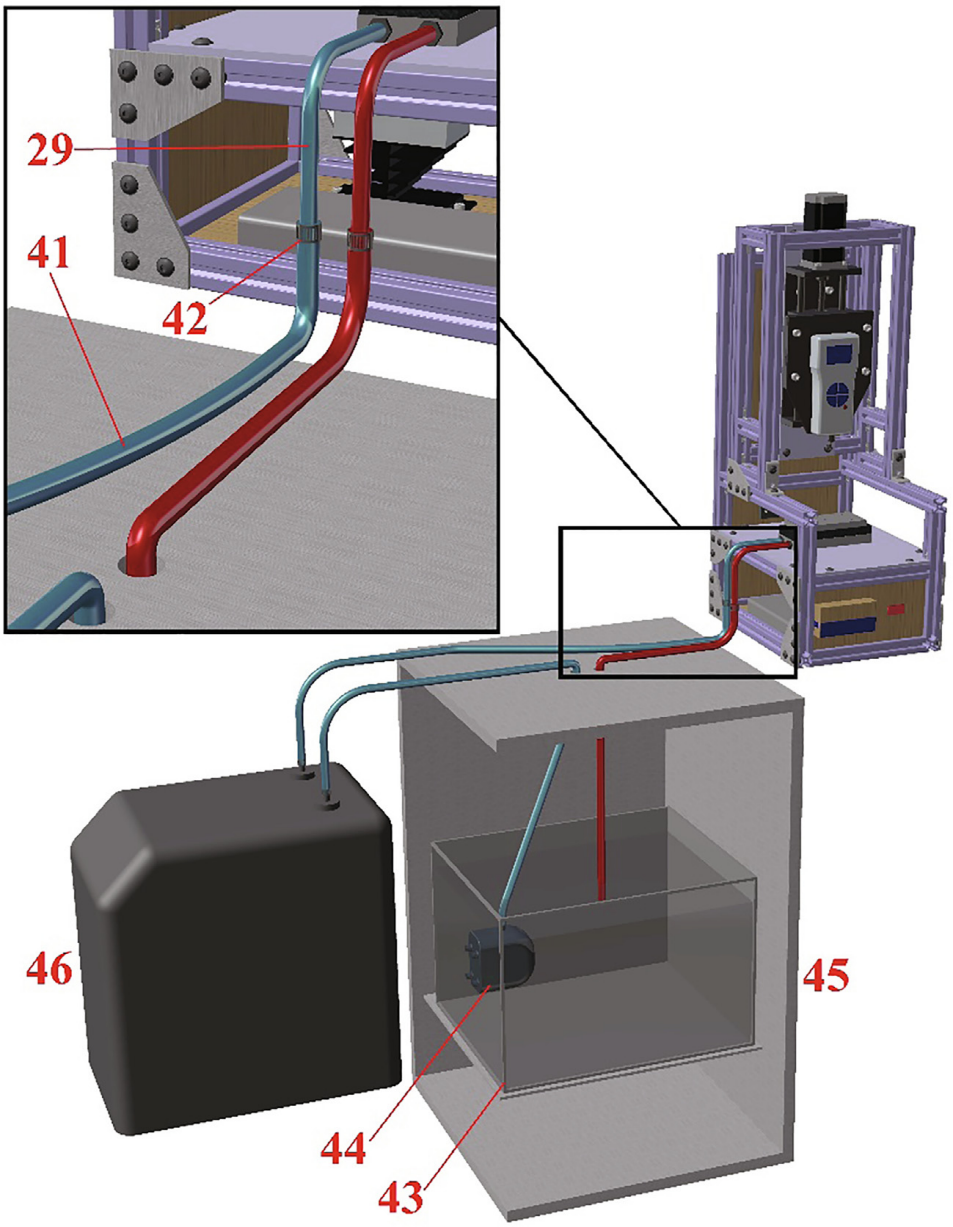
Closed-loop water circulation system via the measurement stage structure, mini fridge, and chiller.

In Table 2, all files required for building the tensile ice adhesion testing apparatus are listed. Each part is named based on the corresponding numbers for components in Fig. 4, Fig. 5, Fig. 6, Fig. 7, Fig. 8, Fig. 9, Fig. 10, Fig. 11 (*i.e.* Part 1 refers to the component numbered as 1 in Fig. 4). These models are provided as IGS files which can be opened in any CAD software. A brief description for other CAD models is explained below in Sec. 2.2 - 2.8.

**Table 2:** Summary of the design files.

Design file name	File type	Open source license	Location of the file
*Designed Ice Holder (R = 5 mm, R = 7.5 mm)*	*STL file*	CC BY-SA 3.0	https://doi.org/10.17632/fp27n5n88v.4#file-847ca78d-d3f0-4856-ba03-865f18385c3b https://doi.org/10.17632/fp27n5n88v.4#file-b482e1a2-e300-4602-a5a4-1a3bf3686e11
*Designed Ice Holder (R = 5 mm)*	*IGS file*	CC BY-SA 3.0	https://doi.org/10.17632/fp27n5n88v.4#file-a1b1b16a-c8f2-408e-afee-6f05e04d77e4
*Normal Ice Adhesion Setup*	*IGS file*	CC BY-SA 3.0	https://doi.org/10.17632/fp27n5n88v.4#file-8b522ffd-e030-41a3-b3c3-595cdd339fe2
*Peltier Plate Stage*	*IGS file*	CC BY-SA 3.0	https://doi.org/10.17632/fp27n5n88v.4#file-7f963416-74e3-4ac9-8baf-ddda9c5d8a99
*Crosshead Assembly*	*IGS file*	CC BY-SA 3.0	https://doi.org/10.17632/fp27n5n88v.4#file-ce5515e0-089c-4e26-b4a6-f11291579d9e
*Movable Structure Assembly*	*IGS file*	CC BY-SA 3.0	https://doi.org/10.17632/fp27n5n88v.4#file-ca422346-0143-4f1e-86c9-a6f0c18ca77b
*Stationary Structure*	*IGS file*	CC BY-SA 3.0	https://doi.org/10.17632/fp27n5n88v.4#file-c0f0de11-0785–4129-ba32-0fd877bd7380
*Water Cooling System*	*IGS file*	CC BY-SA 3.0	https://doi.org/10.17632/fp27n5n88v.4#file-3c21f0c6-ab03-4392-aa27-06b192cc9781
*Part 1*	*IGS file*	CC BY-SA 3.0	https://doi.org/10.17632/fp27n5n88v.4#file-0c80e998-96ba-499c-8119-be0558ad5a41
*Part 2*	*IGS file*	CC BY-SA 3.0	https://doi.org/10.17632/fp27n5n88v.4#file-ce94e828-82c5-4762-a7d5-5cf157f65d88
*Part 3*	*IGS file*	CC BY-SA 3.0	https://doi.org/10.17632/fp27n5n88v.4#file-3ca89f31-d0e0-41c2-b6a1-e95e9bb90025
*Part 4*	*IGS file*	CC BY-SA 3.0	https://doi.org/10.17632/fp27n5n88v.4#file-5d3ff548-9a20-4b03-a31e-420c82049e94
*Part 5*	*IGS file*	CC BY-SA 3.0	https://doi.org/10.17632/fp27n5n88v.4#file-669db276-79c0-48df-b655-81cfaeb7bbcf
*Part 6*	*IGS file*	CC BY-SA 3.0	https://doi.org/10.17632/fp27n5n88v.4#file-24ad39cb-896d-47c4-ba1b-b8ebf76cbd9c
*Part 7*	*IGS file*	CC BY-SA 3.0	https://doi.org/10.17632/fp27n5n88v.4#file-698e8383-99e2-4fbd-849a-44acfe3912f2
*Part 8*	*IGS file*	CC BY-SA 3.0	https://doi.org/10.17632/fp27n5n88v.4#file-c29cd2e3-14fb-4079-92b9-904bd8a237b3
*Part 9*	*IGS file*	CC BY-SA 3.0	https://doi.org/10.17632/fp27n5n88v.4#file-f186890e-bc74-470c-ab28-add4f246102d
*Part 10*	*IGS file*	CC BY-SA 3.0	https://doi.org/10.17632/fp27n5n88v.4#file-a852e3a3-ca2f-4dc6-90f9-f28b3f23ec78
*Part 11*	*IGS file*	CC BY-SA 3.0	https://doi.org/10.17632/fp27n5n88v.4#file-06a309e4-7c86-4550-a399-be4490459047
*Part 12*	*IGS file*	CC BY-SA 3.0	https://doi.org/10.17632/fp27n5n88v.4#file-bfef99b8-ca54-47ee-b396-2157c76a6391
*Part 13*	*IGS file*	CC BY-SA 3.0	https://doi.org/10.17632/fp27n5n88v.4#file-60f119b5-353d-43f4-929a-3fb2f690e3a8
*Part 14*	*IGS file*	CC BY-SA 3.0	https://doi.org/10.17632/fp27n5n88v.4#file-b48e8e43-16a1-4edc-a631-544fda443327
*Part 15*	*IGS file*	CC BY-SA 3.0	https://doi.org/10.17632/fp27n5n88v.4#file-5137fd21-6ef1-4d0f-b589-ce1d451bd2a3
*Part 16*	*IGS file*	CC BY-SA 3.0	https://doi.org/10.17632/fp27n5n88v.4#file-70cf7e33-b7ae-4cba-80ca-45e435ef72af
*Part 17*	*IGS file*	CC BY-SA 3.0	https://doi.org/10.17632/fp27n5n88v.4#file-3cae2cbc-43b2-4715-979c-4e5aa9fa637b
*Part 18*	*IGS file*	CC BY-SA 3.0	https://doi.org/10.17632/fp27n5n88v.4#file-5477e453-6de8-416c-bac5-66d4d7a0cda5
*Part 19*	*IGS file*	CC BY-SA 3.0	https://doi.org/10.17632/fp27n5n88v.4#file-0dde3046-7349-440e-8468-85e68a4e745e
*Part 20*	*IGS file*	CC BY-SA 3.0	https://doi.org/10.17632/fp27n5n88v.4#file-cc8c1c4b-bdae-42b0-818d-84f72ce6bce9
*Part 21*	*IGS file*	CC BY-SA 3.0	https://doi.org/10.17632/fp27n5n88v.4#file-c44b8c6d-cf7f-4c7d-a99a-328518218413
*Part 22*	*IGS file*	CC BY-SA 3.0	https://doi.org/10.17632/fp27n5n88v.4#file-527bd005-d414-47d3-aafe-9c8b7694898e
*Part 23*	*IGS file*	CC BY-SA 3.0	https://doi.org/10.17632/fp27n5n88v.4#file-a46af728-f2ca-4bbd-bd15-17ce61046b6b
*Part 24*	*IGS file*	CC BY-SA 3.0	https://doi.org/10.17632/fp27n5n88v.4#file-a21a3b44-9a7c-47b5-b4d9-15d1706ebceb
*Part 25*	*IGS file*	CC BY-SA 3.0	https://doi.org/10.17632/fp27n5n88v.4#file-16c05f46-ba3d-469e-b6d1-9d8c7d118939
*Part 26*	*IGS file*	CC BY-SA 3.0	https://doi.org/10.17632/fp27n5n88v.4#file-c788db24-65df-4148-9ba8-64d89187128b
*Part 27*	*IGS file*	CC BY-SA 3.0	https://doi.org/10.17632/fp27n5n88v.4#file-0161aac8-36a2-4edc-8593-9810d048e2a0
*Part 28*	*IGS file*	CC BY-SA 3.0	https://doi.org/10.17632/fp27n5n88v.4#file-ca1134ad-a392-4ed3-91c0-d0d6ab8c86c2
*Part 32*	*IGS file*	CC BY-SA 3.0	https://doi.org/10.17632/fp27n5n88v.4#file-deec040e-bac1-4d5c-9000-6feb90723fb1
*Part 34*	*IGS file*	CC BY-SA 3.0	https://doi.org/10.17632/fp27n5n88v.4#file-30f0224b-52b8-415a-a6cc-df94fa68a2b0
*Part 35*	*IGS file*	CC BY-SA 3.0	https://doi.org/10.17632/fp27n5n88v.4#file-f9813b80-6c03-4c71-9ceb-3354f329f519
*Part 37*	*IGS file*	CC BY-SA 3.0	https://doi.org/10.17632/fp27n5n88v.4#file-5be62c0d-c60a-4145-a15b-018e871a6451
*Part 39*	*IGS file*	CC BY-SA 3.0	https://doi.org/10.17632/fp27n5n88v.4#file-ec0ecd83-2e01-4fd5-860b-1fbfad3043d2
*Part 42*	*IGS file*	CC BY-SA 3.0	https://doi.org/10.17632/fp27n5n88v.4#file-affb579e-b2c7-415e-b7fc-5962b861a2c8
*Part 43*	*IGS file*	CC BY-SA 3.0	https://doi.org/10.17632/fp27n5n88v.4#file-5b0f09ed-22b1-436e-909d-cf1f2504cc67
Software	*INO file*	CC BY-SA 3.0	https://doi.org/10.17632/fp27n5n88v.4#file-cd209b96-3ca5-485e-aad9-76727e0bb48f

**Table 3:** Summary of bill and components.

Designator	Component	Number	Cost per unit (USD)	Total cost (USD)	Link	Material type
*Parts 1, 2, 3, 15, 19*	1.00″ x 1.00″ Smooth Surface T-Slotted Profile - Four Open T-Slots, 72″ bars	4	$14.90	$59.60	https://www.tnutz.com/product/ex-1010/	Metal
*Part 4*	Corner Concealed Brackets	46	$1.50	$69.00	https://www.tnutz.com/product/hc-010-a/	Metal
*Part 5*	1/4–20 Slide-in Economy T-Nut - Centered Thread	56	$0.12	$6.72	https://www.tnutz.com/product/et-010–1-4–20/	Metal
*Parts 6, 38*	NEMA 23 425 oz-in Stepper Motor & Driver	1	$57.00	$57.00	https://www.omc-stepperonline.com/1-axis-stepper-cnc-kit-30nm425ozin-nema-23-stepper-motor-and-driver-1-dm542t-23hs45.html	Other
*Part 7*	NEMA 23 WIDE Z axis CNC Slide	1	$160.00	$160.00	https://cnc4newbie.com/store/en/z-axis-slider/z-axis-nema-23-wide-p74c37/	Other
*Part 8*	Black-Oxide Alloy Steel Socket Head Screw, 10–32 Thread Size, 1/2″ Long (Pack of 100)	4	$0.10	$10.07	https://www.mcmaster.com/91251A342	Metal
*Parts 9, 13, 26*	Multipurpose 6061 Aluminum Sheet, 0.19″ Thick, 12″ x 12″	235.12	$0.18	$106.38	https://www.mcmaster.com/89015 K31	Metal
*Part 10*	*Black-Oxide Alloy Steel Socket Head Screw, 5/16″-18 Thread Size, 1″ Long* (Pack of 50)	8	$0.23	$11.48	https://www.mcmaster.com/91251A583	Metal
*Part 11*	Medium-Strength Steel Serrated Flange Locknut, Grade 5, Zinc-Plated, 5/16″-18 Thread Size (Pack of 100)	8	$0.11	$11.03	https://www.mcmaster.com/96282A102	Metal
*Part 12*	Nextech 500 N Force gauge	1	$220.00	$220.00	https://nextechsales.com/collections/force-testing/products/dfs50?variant = 10439792164921	Electronics
*Part 14*	Black-Oxide Alloy Steel Socket Head Screw, M5 × 0.8 mm Thread, 8 mm Long (Pack of 100)	2	$0.10	$9.81	https://www.mcmaster.com/91290A222	Metal
*Part 16*	10 Series 3 Hole - Inside Corner Bracket	2	$1.75	$3.50	https://www.tnutz.com/product/cb-010-c/	Metal
*Part 17*	10 Series 4 Hole − 90°Angled Flat Plate	10	$2.60	$26.00	https://www.tnutz.com/product/jp-010-g/	Metal
*Part 18*	1/4–20 × 0.375″ Flanged Button Head Socket Cap Screw (FBHSCS)	56	$0.07	$3.92	https://www.tnutz.com/product/imperial-button-head/	Metal
*Parts 20, 21, 22, 23, 24,*	1/4″ MDF sheet	1	$2.84	$2.84	https://www.homedepot.ca/product/cutler-group-standard-hardboard-panel-1–4-inches-x-2-feet-x-2-feet/1000434567	Other
*Part 27*	Peltier plate	1	$388.64	$388.64	https://www.mouser.ca/ProductDetail/Laird-Thermal-Systems/DL-120–24-00–00–00?qs = sGAEpiMZZMtAhbGwPnfBjVn2Cl%252Bi6LjjMkPVLovpnu0%3D	Electronics
*Part 28*	1/4″ Barb, 1/4, Nylon Single Barbed Tube Adapter - Black, Male	2	$6.49	$12.98	https://www.mscdirect.com/product/details/48712285	Polymer
*Part 34*	Black-Oxide Alloy Steel Socket Head Screw, M3 × 0.5 mm Thread, 20 mm Long (Pack of 100)	4	$0.11	$10.86	https://www.mcmaster.com/91290A123	Metal
*Part 35*	Black-Oxide Steel Hex Nut, Medium-Strength, Class 8, M3 × 0.5 mm Thread (Pack of 100)	4	$0.09	$9.43	https://www.mcmaster.com/90593A001	Metal
*Part 37*	Black-Oxide Alloy Steel Socket Head Screw, M4 × 0.7 mm Thread, 15 mm Long (Pack of 5)	10	$2.15	$21.48	https://www.mcmaster.com/91290A306	Metal
*Part 39*	Left-Hand Threaded Medium-Strength Steel Hex Nut, Class 5, Zinc-Plated, M4 × 0.7 mm Thread (Pack of 25)	10	$0.23	$5.83	https://www.mcmaster.com/93695A125	Metal
*Part 42*	3/8 in. Stainless Steel Hose Clamps, 10 Pack	1	$5.35	$5.35	https://www.princessauto.com/en/detail/3–8-in-stainless-steel-hose-clamps-10-pk/A-p8641896e	Metal
*Part 43*	Advance Pet Products Heavy Stainless Steel Flat Side Bucket, 6-Quart	1	$14.63	$14.63	https://www.amazon.ca/gp/product/B005GWUR2S/	Other
*Part 29*	1/4 in. Clear PVC Tubing, NSF, Sold by the Foot	10	$0.35	$3.5	https://www.princessauto.com/en/detail/1–4-in-clear-pvc-tubing-nsf-sold-by-the-foot/A-p8576258e	Polymer
*Part 30*	PID controller (AC powered, DC signal)	1	$35.06	$35.06	http://www.auberins.com/index.php?main_page = product_info&products_id = 14	Electronics
*Part 31*	DFRobot DFR0009 LCD Shield for Arduino	1	$17.30	$17.30	https://www.amazon.ca/DFRobot-DFR0009-LCD-Shield-Arduino/dp/B006D903KE/	Electronics
*Part 33*	SODIAL(R) 5 Pcs Dual Row 4 Position Covered Screw Terminal Block Strip 600 V 15A	2	$1.62	$8.13	https://www.amazon.ca/gp/product/B00SUVKE98	Polymer
*Part 36*	Solid state relay (DC control, DC output) with heat sink	1	$2.02	$2.02	https://www.ebay.ca/itm/SSR-25DA-SSR-25AA-SSR-25DD-SSR-Solid-State-Relay-Case-Aluminum-Alloy-Heat-Sink/253967745569	Electronics
*Part 40*	Mean Well HLG-150H-20 (110 V to 24 V 150 W, sealed, fanless)	1	$45.80	$45.80	https://www.arrow.com/en/products/hlg-150 h-20/mean-well-enterprises	Electronics
*Part 41*	3/8 in. Clear PVC Tubing, NSF, Sold by the Foot	10	$0.42	$4.21	https://www.princessauto.com/en/detail/3–8-in-clear-pvc-tubing-nsf-sold-by-the-foot/A-p8576266e	Polymer
*Part 44*	Pawfly 400 GPH Submersible Pump UL400 Quiet Indoor Outdoor Water Pump	1	$18.56	$18.56	https://www.amazon.ca/gp/product/B01MZI9MI1	Other
*Part 45*	Danby DAR026A1WDD-6 2.6 Cu.Ft. Mini Fridge	1	$142.82	$142.82	https://www.amazon.ca/Danby-DAR026A1WDD-6-Cu-Ft-Fridge-White/dp/B07TK552ZK	Other
*Part 46*	Active Aqua AACH10HP Water Chiller Cooling System, 1/10 HP	1	$363.44	$363.44	https://www.amazon.ca/gp/product/B07BHHP71C	Other
*Part 55*	10 SERIES – Black Plastic End Cap w/Push-Ins	10	$0.30	$3.00	https://www.tnutz.com/product/ec-010/	Polymer
*Part 47*	Stranded Wire 300 V AC, 16 Wire Gauge (black)	1	$6.98	$6.98	https://www.mcmaster.com/catalog/126/904	Electronics
*Part 48*	Stranded Wire 300 V AC, 16 Wire Gauge (red)	1	$6.98	$6.98	https://www.mcmaster.com/catalog/126/904	Electronics
*Part 49*	7–24 V to 5 V (USB) step-down converter (to power Arduino)	1	$11.11	$11.11	https://www.amazon.ca/gp/product/B00INWXXCO/	Electronics
*Part 50*	100x Blue/Red Insulated Spade Fork Connector Electrical Crimp Wire Terminals - Red	1	$9.06	$9.06	https://www.amazon.ca/gp/product/B01KTTA9VG/	Electronics
*Part 51*	WTK-10–24 Bolt-On Washer Thermocouple Assemblies	1	$10.71	$10.71	https://www.omega.ca/en/sensors-and-sensing-equipment/temperature/sensors/surface-sensors/p/WT	Electronics
*Part 52*	Bergen Industries 3-Wire Appliance and Power Tool Cord, 6′, 16 AWG, 13A/125 V AC, 1625 W, Black	1	$3.19	$3.19	https://www.amazon.ca/Bergen-Industries-3-Wire-Appliance-Power/dp/B07C9D6CXY	Electronics
*Part 53*	Arduino UNO	1	12.99	12.99 USD	https://www.amazon.com/ELEGOO-Board-ATmega328P-ATMEGA16U2-Compliant/dp/B01EWOE0UU/	Electronics
*Part 54*	Jumper wires	1	11.95	11.95 CAD	https://www.amazon.ca/Elegoo-120pcs-Multicolored-Breadboard-arduino/dp/B01EV70C78/	Electronics
*Part 56*	Emma kites 70 ~ 2000 lb Kevlar Kite String Braided	1	$21.39	$21.39	https://www.amazon.ca/gp/product/B00ZPR1BW4/	Other

### 2.2 IGS and STL files for designed ice holder

The Designed Ice Holder.IGS file provides a 3D model of the ice holder which is designed for tensile ice adhesion testing. Users can use the Designed Ice Holder.STL to print the holder on any standard 3D printer. Two sizes are available, with radii of 5 mm and 7.5 mm.

### 2.3 Normal ice adhesion setup

This file contains the assembly model of the tensile ice adhesion testing apparatus. The assembly model provides the mounting positions for each individual part listed in Table 2.

### 2.4 Peltier plate stage

This file provides an assembly model of the Peltier plate stage mounted on the stationary structure and connected to the optional water cooling system.

### 2.5 Crosshead assembly

This file includes the assembly of the crosshead which allows for a uniaxial degree of freedom for the force gauge. Mounting positions for each component of the crosshead assembly are provided to users in this file.

### 2.6 Movable structure assembly

The Movable Structure Assembly.IGS file contains the assembly of the movable structure built of T-slotted framing, which provides translational degrees of freedom for the mounted crosshead assembly.

### 2.7 Stationary structure

The Stationary Structure.IGS file contains the assembly model of the base structure, which the movable structure and Peltier stage are mounted on. Users can find the mounting positions of different components with respect to each other.

### 2.8 Water cooling system

The assembly model of the water cooling system contains a closed-loop water circulation system which connects the Peltier plate to the cooling system, including a water chiller and mini-fridge. The length of tubing shown in this file is arbitrary and may differ based on the positions of the chiller, fridge, and testing apparatus.

## 3 Bill of materials

The total price to build the exact device comes around $2,000 USD (Table 3). However, this price includes the closed-loop water circulation system, which increases electricity consumption and cost, but reduces water waste. Alternatively, the Peltier stage may be water cooled simply by connecting the PVC tubes to a sink. In this case the mini-fridge, bucket, submersible pump, additional PVC tubing, and the chiller are not needed, reducing the cost to $1,450.

### 3.1 Tools needed

• Philips head and flat head screw drivers
• Alan keys/hex wrenches
• Soldering gun and solder
• Glue/strong adhesive (3 M scotch-weld adhesive DP604NS and 3 M 9629PC double-sided tape recommended

## 4 Build instructions

This device consists of four major components (Fig. 2): (1) The stationary structure on which the electronics are attached and which provides the mechanical support of the system. (2) The movable structure which is fixed to the stationary structure while having the freedom to move in both x- and y- directions, and supports and holds the crosshead. (3) The crosshead, which is mainly comprised of a motor and a force gauge with a freedom of movement in the z-direction, due to the linear motion stage. And (4) the Peltier stage which is fixed on the stationary structure and is responsible for the cooling. Below, step-by-step build instructions are given along with figures with numbers for easy understanding. Table 4 includes the number directory.

**Table 4:** Building part directory.

Part name	Number
Smooth Surface T-Slotted Profile- 16 in. Long	1
Smooth Surface T-Slotted Profile- 8 in. Long	2
Smooth Surface T-Slotted Profile- 3 in. Long	3
Corner Concealed Brackets	4
1/4–20 Slide-in T-Nut- Centered Thread	5
NEMA 23 425 oz-in Stepper Motor	6
NEMA 23 WIDE Z axis CNC Slide	7
Black-Oxide Alloy Steel Socket Head Screw, 10–32 Thread Size, 1/2″ Long	8
Multipurpose 6061 Aluminum Sheet (See Fig. 3c for Dimensions)	9
Black-Oxide Alloy Steel Socket Head Screw, 5/16″-18 Thread Size, 1″ Long	10
Medium-Strength Steel Serrated Flange Locknut, Grade 5, Zinc-Plated, 5/16″-18 Thread Size	11
Nextech 500 N Force Gauge	12
Multipurpose 6061 Aluminum Sheet (See Fig. 3a for Dimensions)	13
Black-Oxide Alloy Steel Socket Head Screw, M5 × 0.8 mm Thread, 8 mm Long	14
Smooth Surface T-Slotted Profile- 12 in. Long	15
3 Hole – Inside Corner Bracket	16
4 Hole − 90°Angled Flat Plate	17
1/4–20 × 0.375″ Flanged Button Head Socket Cap Screw (FBHSCS)	18
Smooth Surface T-Slotted Profile- 24 in. Long	19
MDF sheet 1–352 mm × 205 mm × 6.35 mm	20
MDF sheet 2–60 mm × 205 mm × 6.35 mm	21
MDF sheet 3–100 mm × 205 mm × 6.35 mm	22
MDF sheet 4–280 mm × 205 mm × 6.35 mm	23
MDF sheet 5–100 mm × 205 mm × 6.35 mm	24
Smooth Surface T-Slotted Profile- 11 in. Long	25
Multipurpose 6061 Aluminum Sheet (See Fig. 3b for Dimensions)	26
Peltier Plate	27
1/4 in. Barb, 1/4, Nylon Single Barbed Tube Adapter - Black, Male	28
1/4 in. Clear PVC Tubing, NSF	29
PID Controller (AC powered, DC signal)	30
DFRobot DFR0009 LCD Shield for Arduino	31
Housing for Arduino	32
SODIAL(R) 5 Pcs Dual Row 4 Position Covered Screw Terminal Block Strip 600 V 15A	33
Black-Oxide Alloy Steel Socket Head Screw, M3 × 0.5 mm Thread, 20 mm Long	34
Black-Oxide Steel Hex Nut, Medium-Strength, Class 8, M3 × 0.5 mm Thread	35
Solid State Relay (DC control, DC output) with Heat Sink	36
Black-Oxide Alloy Steel Socket Head Screw, M4 × 0.7 mm Thread, 15 mm Long	37
NEMA 23 425 oz-in Stepper Driver	38
Left-Hand Threaded Medium-Strength Steel Hex Nut, Class 5, Zinc-Plated, M4 × 0.7 mm Thread	39
Mean Well HLG-150H-20	40
3/8 in. Clear PVC Tubing, NSF	41
3/8 in. Stainless Steel Hose Clamps	42
Stainless Steel Flat Side Bucket	43
Submersible Pump	44
Mini Fridge	45
Water Chiller Cooling System	46
Stranded Wire 300 V AC, 16 Wire Gauge (black)	47
Stranded Wire 300 V AC, 16 Wire Gauge (red)	48
7–24 V to 5 V (USB) step-down converter (to power Arduino)	49
100x Blue/Red Insulated Spade Fork Connector Electrical Crimp Wire Terminals	50
WTK-10–24 Bolt-On Washer Thermocouple Assemblies	51
3-Wire Appliance and Power Tool Cord	52
Arduino UNO	53
Jumper wires	54
10 SERIES – Black Plastic End Cap w/Push-Ins	55
Emma kites 70 ~ 2000 lb Kevlar Kite String Braided	56

### 4.1 Structural instructions

Cut and drill the aluminum sheets (9), (13), and (26) with respect to the dimensions depicted in Fig. 3.

*Phase 1 (see* Fig. 4 *):*

1. Slide two T-nuts (5) into a T-slotted profile (2). Do the same for another T-slotted profile (2).
2. Use corner concealed brackets (4) to attach the T-slotted profiles (1), (2) and (3) to each other as shown in Fig. 4. Ensure that the profiles from Step 1 are placed at the back end).

*Phase 2 (see* Fig. 5 *):*

3. Attach the motor (6) to the Z axis CNC slide (7) using four steel socket head screws (8).
4. Attach the Z axis CNC slide (7) from Step 3 to the aluminum sheet (9) using four pairs of steel socket head screws (10) and locknuts (11).
5. Fix the force gauge (12) to the aluminum sheet (13) using two steel socket head screws (14)
6. Attach the aluminum sheet (13) from Step 5 to the Z axis CNC slide (7) from Step 4 using four pairs of steel socket head screws (10) and locknuts (11).

*Phase 3 (see* Fig. 6 *):*

7. Slide three T-nuts (5) into the outer slot and one into the top slot of two T-slotted profiles (15).
8. Slide one T-nut (5) to each of the vertical T-slotted profiles (1) from Phase 1, as illustrated in Fig. 6.
9. Attach T-slotted profiles (15) from Step 7 to the Phase 1 structure from Step 8 using two corner brackets (16), two 90°angled flat plates (17) and twelve Flanged Button Head Socket Cap Screws (FBHSCS) (18).

Note: The structure from Phase 1 should be able to slide freely on T-slotted profiles (15) at this point.

10. Use four FBHSCS (18) and the previously inserted T-nuts in Step 1 to attach the final structure from Phase 2 to the structure from Step 9.
11. Insert four corner concealed brackets (4) to the bottom slots of the T-slotted profiles (15).

*Phase 4 (see* Fig. 7 *):*

12. Begin from the bottom of the structure.
13. Slide T-nuts (5) in the slots of the T-slotted profiles as illustrated in Fig. 7.
14. Insert all corner concealed brackets (4) as illustrated in Fig. 7.
15. Use two T-slotted profiles (2) and two T-slotted profiles (15) to form a frame around the MDF Sheet 4 (23). The MDF sheet should be inserted within the slots of the T-slotted profiles.
16. Slide two horizontal T-slotted profiles (2) into the slots of the two vertically standing T-slotted profiles (19) from the bottom (let them be loose).
17. Slide one T-slotted profile (2) horizontally into the two shorter vertically standing T-slotted profiles (2) from the bottom (let them be loose).
18. Use two T-slotted profiles (15) and corner concealed brackets (4) to connect the tall (19) and short (2) T-slotted profiles.
19. Use the corner concealed brackets (4) to fix the vertically standing T-slotted profiles (2) and (19) in the slots of the bottom structure from Step 14.
20. Insert MDF Sheets 3 (22) and 5 (24) into the slots of the vertically standing T-slotted profiles as shown in Fig. 7.
21. Insert the MDF sheets from the top with the horizontal T-slotted profiles inserted in previous steps and fix the structure.
22. Insert the MDF Sheet 2 (21) into the slots of the tall T-slotted profiles (19), but below the second horizontal T-slotted profile (2).
23. Fix the second horizontal T-slotted profile (2) on top of the MDF sheet by inserting the sheet into the slots.
24. Slide MDF Sheet 1 (20) into the slots of the tall T-slotted profiles (19).
25. Fix a T-slotted frame (2) on top MDF Sheet 1 using corner concealed brackets (4).
26. Fix the whole structure from two sides usinga 90° angled flat plate (17) and FBHSCS (18).

Note: Now all the components of this structure may now be strongly tightened and should be without any free movement.

*Phase 5 (see* Fig. 8 *):*

27. Use screws (37) and (34) and nuts (39) and (35) to attach the electronics to the MDF sheets of the structure from Phase 4, as illustrated in Fig. 8
28. Attach PID controller (30) to MDF Sheet 5 (24).
29. Attach the LCD shield for Arduino (31) to the housing (32).
30. Glue the housing to the MDF Sheet 5.

STOP! At this point the user may want to skip to the Electronics Instructions below, as at this point there is maximum access to all mounted electronics components.

*Phase 6 (see* Fig. 9 *):*

31. Attach Nylon single barbed tube adapter (28) to the Peltier plate (27).
32. Use eight FBHSCS (18) and the T-nuts inserted in Step 13 to fix aluminum sheet (26) as shown in Fig. 9.
33. Remove the 3 internal screws on the bottom of the Peltier plate (27). Fix the Peltier plate (27) on the aluminum sheet (26) by re-inserting its three internal screws into the 3 central holes of (26).
34. Attach the PVC tubes (29) to the Nylon single barbed tube adapter (28).
35. Attach and fix the movable structure to the stationary structure now containing the Peltier assembly, as shown in Fig. 10.

Though not necessary, the ends of all T-slotted profiles may be capped with End Caps (55) if there remains any sharp edges from cutting.

*Phase 7 (see* Fig. 11 *):*

36. Attach PVC tube (41) to (29) using a stainless-steel hose clamp (42).
37. Attach the submersible pump (44) to the stainless-steel bucket (43).
38. Fill the stainless-steel bucket (43) with water and place inside the mini fridge (45).
39. Connect the Peltier plate system, chiller cooling system (46) and submersible pump (44) using the PVC tubes (41) to create a closed-loop water circulation system.

### 4.2 Electronics instructions

**Fig. 12:**
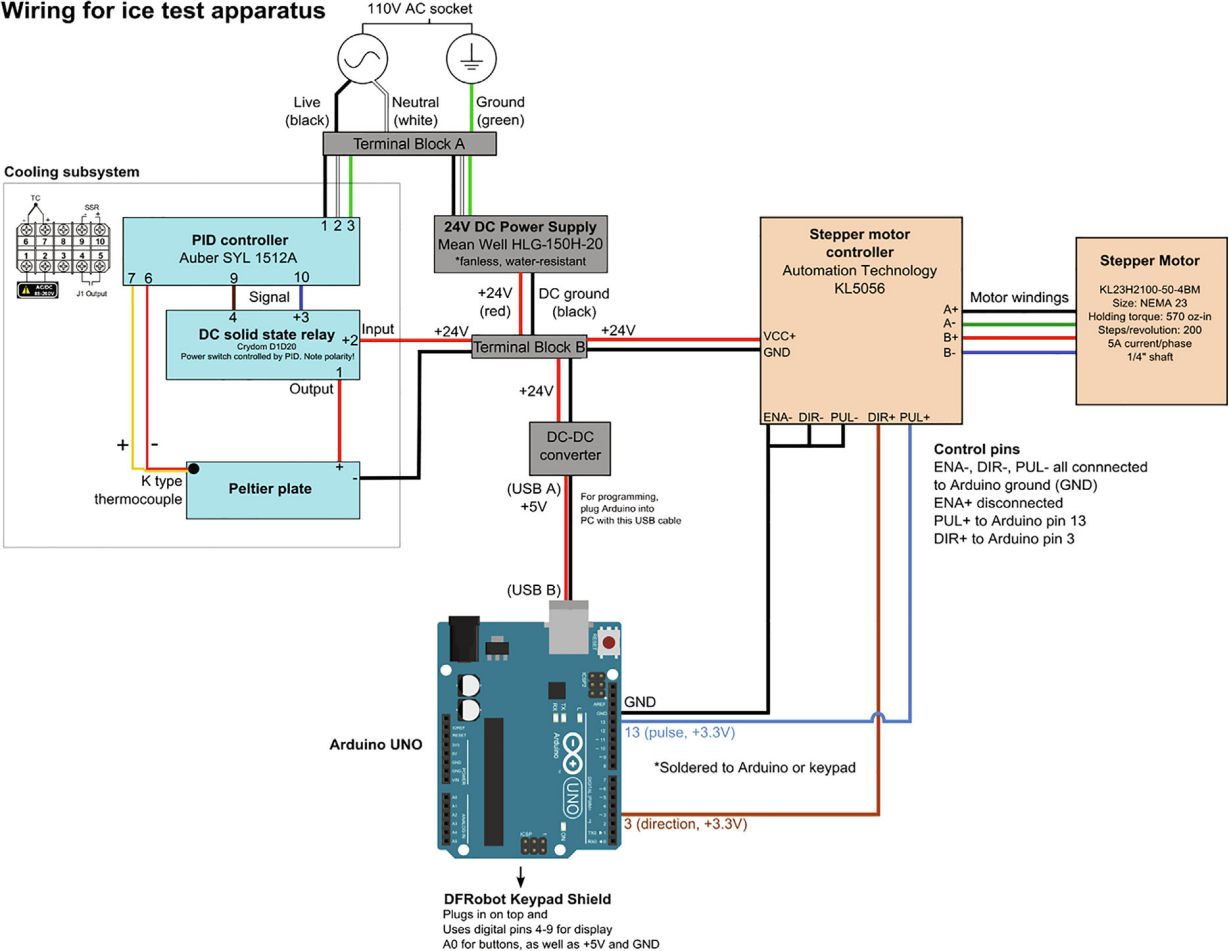
Wiring diagram for the electronics of the tensile ice adhesion measurement device.

The overall wiring diagram is shown in Fig. 12 below. Individual instructions follow.

### 4.3 Power socket

• The entire instrument is powered by a Type B power plug (52) connected to a wall power socket that has 110 V 60 Hz power. Connect the exposed positive, negative, and ground terminals to Terminal Block A (33) on separate ports using fork connectors (50).
• Connect the power supply (40) to the power plug through Terminal Block A (positive to positive, neutral to neutral, negative to negative) with fork connectors (50) using the stripped wires emanating from the power supply.

### 4.4 Arduino

• Carefully mount the Keypad Shield (31) on top of the Arduino UNO (53). The shield uses pins 4–9 for the display, A0 for buttons, and the +5 V and GND pins.
• Connect ports GND, 13, and 3 of Arduino UNO (53) to ports (ENA-, DIR-, PUL-), DIR+, and PUL+ of the stepper motor controller (6), respectively, using jumper wires (54). Solder these on the back of the Arduino as the Keypad Shield (31) sits on top of Arduino.
• Connect the Arduino UNO to the DC-DC step down converter (49), which lowers the +24 V from the Power Supply to +5 V. Use the USB cable (type A-Male to B-Male) included in the Arduino UNO kit. Insert the USB type B male connector into the Arduino UNO and the type A male into the USB port of the DC-DC converter.
• Connect the positive and negative terminals of the DC-DC converter to the positive and negative terminals of the power supply through Terminal Block B (33) with fork connectors (50).

### 4.5 Peltier and temperature control

• Connect ports 1, 2, and 3 of the PID controller (30) to the positive, neutral, and negative terminals, respectively, of the power plug via Terminal Block A. Reuse the same terminals that the power plug is already using by loosening the clamp screw, adding the additional wire, and then tightening.
• Connect ports 9 and 10 of the PID controller to ports 3 and 4 of the solid state relay (36), respectively, with stripped wires (47,48) and fork connectors (50).
• Connect the stripped red wire (positive terminal) coming out of the Peltier plate (27) to port 1 of the solid state relay (36) with fork connectors (50).
• Connect port 2 of the solid state relay (36) to the positive terminal of the power supply (40) through Terminal Block B using a stripped wire and fork connector.
• Connect the negative terminal (black wire) of the Peltier plate (27) to the negative terminal of the power supply through Terminal Block B using a fork connector and stripped wire.
• Connect the positive and negative terminals of the Type-K thermal couple (51) to ports 7 and 6 of the PID controller, respectively.
• Mount the thermal couple on the Peltier plate with a screw (14).

### 4.6 Motor and motor controller

• Connect the VCC+ and GND terminals of the stepper motor controller (6) to the positive and negative terminals of the power supply, respectively, through Terminal Block B using stripped wires and fork connectors. Reuse the same terminals that the power supply is already using by loosening the clamp screw, adding the additional wire, and then tightening.
• The stepper motor (6) comes with black, green, red, and blues wires emanating from it. Connect the stepper motor controller ports A+ to black, A- to green, B+ to red, and B- to blue of the stepper motor.

## 5 Operation instructions

### 5.1 Hardware instructions

Before using the system, the ice holders should be 3D-printed out of a desired resin. PLA printed very well and was sufficiently hydrophobic in order to prevent leakage. For each printed ice holder, thread a ~ 20 cm long piece of kite string (56) through the hole at the top of the ice holder and tie the two ends in a knot so that a loop is formed.

• Turn the system on either by plugging the power cord into an outlet, or turning on a surge protector if using one. Set the temperature as preferred using the PID controller. Calibrate per manual instructions if using for the first time.
• Set the pull-off speed (Slow Forward/Reverse) using the Arduino (see 5.2 below for details). The UNO remembers the last used configuration, so skip this step if not changing the speed from a prior measurement.
• Turn off the device to place the test material and the ice holders. It is important to strongly fix the test material on the Peltier stage in order to prevent deflection and bending during tension. When the expected tensile forces are low, substrates can be fixed to the Peltier stage using 3 M 9629PC double-sided tape. If higher forces are expected, or if the materials exhibit poor tape adhesion, the materials can be glued to aluminum foil using an appropriate adhesive (3 M scotch-weld adhesive DP604NS recommended) and then the foil taped to the Peltier stage using the 3 M 9629PC double-sided tape.
• Clean the surface using isopropyl alcohol, acetone, or any solvent suitable for the substrate material.
• Place the ice holders on the surface with a clearance of at least 1 mm from each other and the edges of the substrate.
• Inject de-ionized water into the ice holders from the openings in its upper surface using a syringe or pipette, until the cylinder is completely full. The 1 cm diameter ice holders require ~ 0.785 mL whereas the 15 cm diameter ice holders require ~ 1.766 mL. Make sure no leaking occurs from the bottom of the holders as it will affect the measured tensile force.
• Turn the water circulation system (submersible pump) and the device on at the same time. The temperature of the cold stage will begin to drop to the pre-set temperature. Freezing occurs directionally from the bottom of the holder contacting the cold sample surface.
• Wait until the water inside the holders is completely frozen and forms glaze (also known as bulk water ice). To verify that the ice has fully frozen, poke the ice through the opening on the upper side of the ice holders with a syringe tip.
• Turn on the force gauge and align the hook manually, exactly above any holder. Place the looped kite string around the force gauge hook.
• Use the Fast Forward/Reverse buttons on the LCD shield to position the hook end far enough from the holder such that the kite string is not under tension but there is minimal amount of slack in the cord.
• Turn on the force gauge or, if already on, zero any residual measurements. Push the Slow Reverse button to pull the ice holder off of the substrate at the pre-set rate, until complete detachment occurs. Record the measured force.
• Repeat the previous 3 steps for all additional ice holders.
• In order to remove the material from the stage, detach it while the stage remains at sub-zero temperatures, as the tape comes off much easier.
• Clean up the tape residue from the Peltier stage before running a second test.
• To turn off the machine simply unplug the power cords.

### 5.2 Software instructions

The Arduino code allows the user to move the force gauge up and down at two different speeds: SLOW FORWARD, FAST FORWARD, SLOW REVERSE, and FAST REVERSE. The FAST speeds are set by the variable maxRate in the code (Line 34), which gives a suitably high speed to quickly move the gauge up and down. The SLOW rate is programmable using the LCD shield buttons. The linear stage moves by the rotation of the motor, which corresponds to a certain distance (*i.e.* rpm is converted to linear velocity). For part (6) this corresponds to 0.47 µm/sec, although the user should verify this amount manually after install. In the Arduino code, this value is held in the variable linConv (Line 38). The string on Line 135 should also be adjusted if this value is changed.

To change the speed, press SELECT to enter the velocity selection screen. The selected number will be a multiple of linConv, and the digit selected can be cycled using the LEFT (SLOW REVERSE) and RIGHT (SLOW FORWARD) buttons. The value can be cycled using the UP (FAST FORWARD) and DOWN (FAST REVERSE) keys. Once a suitable multiple of linConv is chosen, press SELECT again to exit the menu. For example, 100.11 µm/sec corresponds to 213 as 0.47 µm/sec × 213 = 101.11 µm/sec.

The four directional buttons are all binary ON/OFF. For example, to move down at the selected velocity, press SLOW FORWARD. To stop the instrument, press SLOW FORWARD again. While moving, only the currently pressed button can stop the instrument. Caution should therefore be used when pressing FAST FORWARD and FAST REVERSE, as the end of the linear stage could be reached and this could damage the instrument or substrate.

## 6 Validation and characterization

In order to validate the performance of the system, the tensile ice adhesion, *σ*_ice_, of aluminum, stainless steel, and brass were first measured. Next, the performance of the device was characterized with respect to different test parameters such as temperature, pull-off speed, ice/substrate interfacial area, and thickness of the substrate. To evaluate these parameters the ice/surface fracture needs to be adhesive (rather than cohesive failure through the ice itself), so polyethylene (PE) and polypropylene (PP) sheets were used as they are known to exhibit lower ice adhesion than metals [29]. At least 10 measurements were recorded for each sample to verify the system’s repeatability.

### 6.1 Materials

Polypropylene, polyethylene, aluminum, brass, and stainless-steel sheets were purchased from McMaster-Carr.

### 6.2 Results

Similar to their adhesion to ice under shear loading [30], [31], cohesive rather than adhesive fracture was observed between ice and the three metals tested. The tensile ice adhesion of aluminum, stainless steel, and brass at −15 °C were *σ*_ice_ = 1,400 ± 300 kPa, 1,300 ± 400 kPa, and 1,200 ± 200 kPa, respectively (Table 5). The measured tensile ice adhesion strength for aluminum fell within the range of reported literature values [11], [32], [28]. Literature values for steel and brass were not available.

**Table 5:** Tensile ice adhesion strength of different materials using a pull-off speed of 100 µm/s, an interfacial area of 0.785 cm^2^, and a test temperature of −15 °C.

Material	Thickness (mm)	*σ*_ice_ (kPa)	Fracture type
Polypropylene (PP)	3.251	460 ± 120	Adhesive
Polyethylene (PE)	3.251	270 ± 80	Adhesive
Aluminum	0.076	1400 ± 300	Cohesive
Brass	0.076	1200 ± 200	Cohesive
Stainless Steel	0.076	1300 ± 400	Cohesive

**Fig. 13:**
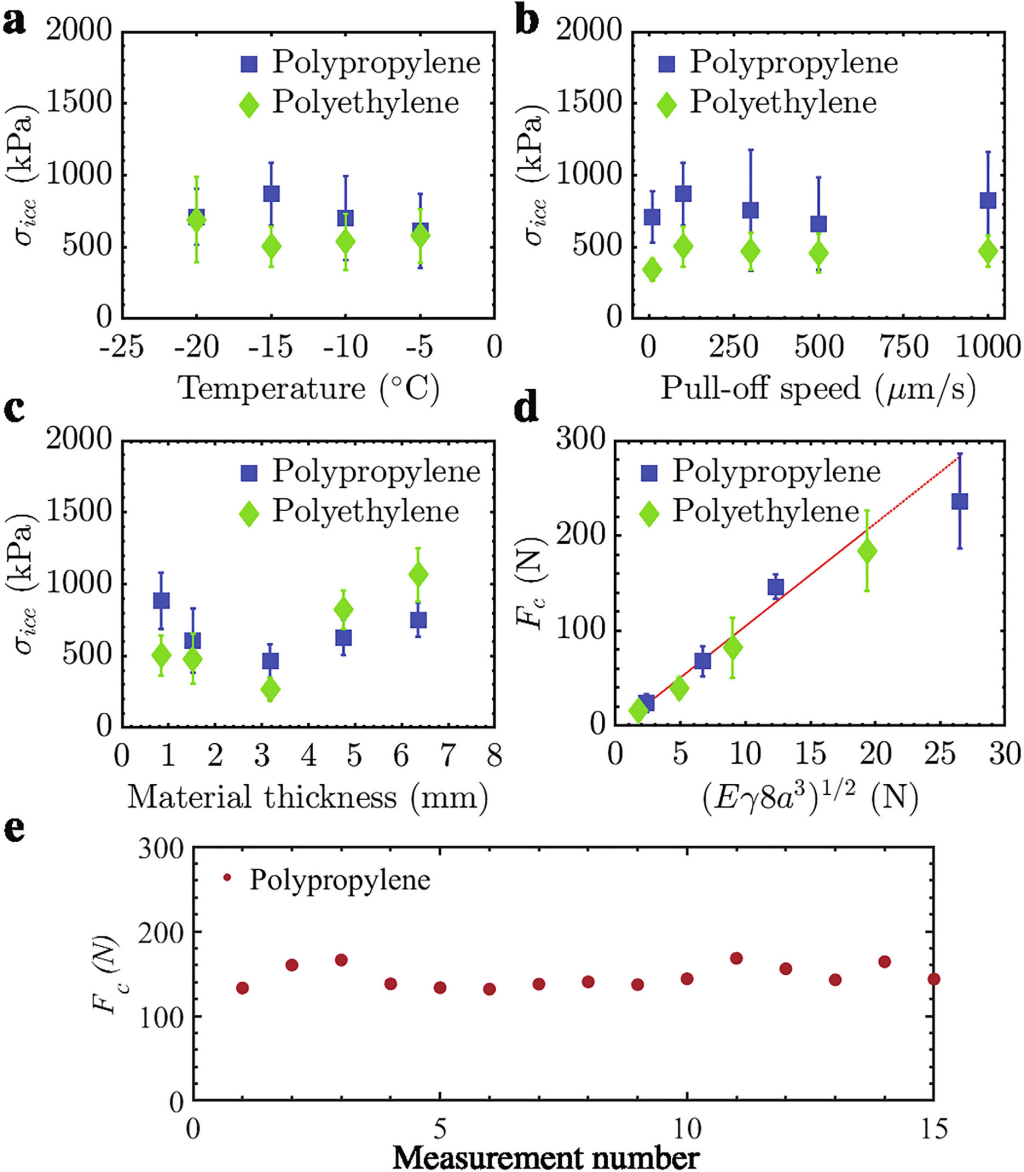
Tensile ice adhesion strength of polypropylene and polyethylene as a function of (a) temperature, (b) pull-off speed, and (c) thickness of the material. (d) Critical detachment force for different radii of ice, plotted according to Eq. (1). (e) Fifteen individual measurements of the critical force to detach ice from polypropylene at −15 °C. The interfacial area was 1.77 cm^2^.

The measured tensile adhesive fracture of PP and PE with ice, while varying the test parameters, was used to determine the precision and performance of the device. First, the temperature of the stage was varied from −5 °C to −20 °C in 5 °C increments while keeping other test variables constant (pull-off speed = 100 µm/s, area of ice, *A* = 0.785 cm^2^, material thickness = 0.84 mm). The tensile ice adhesion of PP and PE were independent of temperature within the range tested (Fig. 13a), similar to prior studies of tensile ice adhesion on other materials [13], [14].

Next, the rate of the applied tensile force was varied between 10 µm/s – 1000 μm/s at a test temperature of −15 °C and the same interfacial area and thickness of the underlying material. The tensile ice adhesion strengths of PP and PE were also found to be independent of the rate of the applied force within this range of velocity (Fig. 13b).

The tensile ice adhesion strength of PP and PE sheets of varying thickness was also measured at −15 °C while maintaining a pull-off speed of 100 µm/s and an interfacial area of 0.785 cm^2^. A measurable but statistically insignificant decrease in tensile ice adhesion was observed by increasing the substrate thickness to 3.2 mm, followed by an equally statistically insignificant increase, up to a thickness of 6.2 mm (Fig. 13c). The results from varying the temperature, pull-off speed, and material thickness were all statistically equivalent, indicating that the device was quite precise for the ranges of variables evaluated.

The adhesion of a rigid stud to an incompressible elastic solid has been previously shown to depend on stud size [33], [34], [35]. As such, it was expected that the interfacial area of ice would alter the observed values of *σ*_ice_. From Kendall’s original formulation, the critical load to de-bond a circular stud, *F_c_*, is given by [35],

(1) $$F_{c}=X\sqrt{E\gamma ({2a)}^{3}}$$

where *E* is the elastic modulus, *γ* is the interfacial surface energy, 2*a* is the stud diameter, and *X*  = π/(1 – *ν*^2^), *ν* being the Poisson ratio. Accordingly, a plot of *F*_c_ against [*Eγ(*2*a)*^3^]^1/2^ should fall on a straight line. The recorded tensile ice adhesion measurements when varying the iced area for PP and PE followed the above relation well, as shown in Fig. 13d. More than any other measurements, this dataset indicated the device was working correctly and with high precision, as the adhesion measurements matched known laws of fracture mechanics precisely.

The reproducibility of the ice type and repeatability of the interfacial fracture process was studied by monitoring the critical force to detach ice on the 0.84 mm thick PP with an interfacial area of 1.77 cm^2^ at −15 °C and pull-off speed of 100 µm/s (Fig. 13e). The small scatter of the 15 independent data points highlighted the instrument’s precision, and was on par with other shear [29] and tensile [14] ice adhesion measurement devices.

## Declaration of Competing Interest

The authors declare that they have no known competing financial interests or personal relationships that could have appeared to influence the work reported in this paper.
